# Extremely rapid acclimation of *Escherichia coli* to high temperature over a few generations of a fed-batch culture during slow warming

**DOI:** 10.1002/mbo3.146

**Published:** 2013-12-20

**Authors:** Stéphane Guyot, Laurence Pottier, Alain Hartmann, Mélanie Ragon, Julia Hauck Tiburski, Paul Molin, Eric Ferret, Patrick Gervais

**Affiliations:** 1 UMR A 02.102 Procédés Alimentaires et Microbiologiques, Equipe Procédés Microbiologiques et Biotechnologiques, AgroSup Dijon, Université de Bourgogne1 Esplanade Erasme, 21000, Dijon, France; 2 UMR 1347 Agroécologie – I.N.R.A.17 rue Sully BP 86510, 21065, Dijon Cedex, France

**Keywords:** Acclimation, *Escherichia coli*, slow warming, thermal niche

## Abstract

This study aimed to demonstrate that adequate slow heating rate allows two strains of *Escherichia coli* rapid acclimation to higher temperature than upper growth and survival limits known to be strain-dependent. A laboratory (K12-TG1) and an environmental (DPD3084) strain of *E. coli* were subjected to rapid (few seconds) or slow warming (1°C 12 h^−1^) in order to (re)evaluate upper survival and growth limits. The slow warming was applied from the ancestral temperature 37°C to total cell death 46–54°C: about 30 generations were propagated. Upper survival and growth limits for rapid warming (46°C) were lower than for slow warming (46–54°C). The thermal limit of survival for slow warming was higher for DPD3084 (50–54°C). Further experiments conducted on DPD3084, showed that mechanisms involved in this type of thermotolerance were abolished by a following cooling step to 37°C, which allowed to imply reversible mechanisms as acclimation ones. Acquisition of acclimation mechanisms was related to physical properties of the plasma membrane but was not inhibited by unavoidable appearance of aggregated proteins. In conclusion, *E.coli* could be rapidly acclimated within few generations over thermal limits described in the literature. Such a study led us to propose that rapid acclimation may give supplementary time to the species to acquire a stable adaptation through a random mutation.

## Introduction

To study the effects of thermal changes on organisms, biologists often focus on microorganisms, as reported by Bennett and Hughes (2009). The bacterium *Escherichia coli* appears to be a particularly ideal biological system for the investigation of evolutionary responses to environmental stress. This bacterium possesses the appropriate properties for this type of investigation: a short generation time and specificity of adaptation (Lenski and Bennett 1993; Buckling et al. 2009). For these reasons, this bacterium is generally used to study the behavior of a cell population exposed to moderate or severe increases in temperature (Bennett et al. 1990; Morozov et al. 1997; Bennett and Hughes 2009). The upper thermal limit of survival of a bacterium (or upper limit of the thermal niche) is defined as the maximal temperature at which the cells survived but did not grow whereas, the upper thermal limit of cell growth is defined as the maximal temperature at which the cells increased in number over time. Concerning *E. coli*, these parameters are extremely variable depending upon a lot of parameters and more particularly the number of generations. For instance, Membré et al. (2005) established that the upper limit of survival is 45.6°C (from a study performed on 10 strains) and the optimal growth temperature is 37°C. Nevertheless, some authors as Rudolph et al. (2010) obtained a higher maximum growth temperature of 48.5°C when bacteria were propagated for more than 600 generations. However, some studies have showed that acclimation of *E. coli* to temperatures higher than its upper thermal limit induced a dramatic reduction in bacterial fitness (Bennett and Lenski 1993) and an inhibition of growth (Ron and Davis 1971) could occur.

When exposed to short-term environmental changes, individual organisms may acclimate. Acclimation is a general response, also called “phenotypic plasticity” (Bennett and Lenski 1997; Huey et al. 1999; Garland and Kelly 2006), which has been observed in prokaryotic cells such as bacteria and in multicellular eukaryotic organisms such as *Drosophila* (Huey et al. 1999) and is often thought to enhance or reduce the organism's endurance or fitness in the new environment. In contrast to adaptation (which refers to mutations, i.e., irreversible genetic changes), acclimation does not involve any mutation and refers to nonpermanent changes (as gene overexpression, epigenetic modifications, changes in proteins and in membrane, changes in metabolic rates, etc.; Bowler 2005). These changes are induced by different types of stress, including the chemical stress of starvation and the physical stress of temperature increases (Bennett and Lenski 1997; Stillman 2003; Schwarz and Forchhammer 2005) and could be maintained over few generations (Bennett and Lenski 1997). Indeed, although acclimation is a process that affects an individual organism within its lifetime, it has been established that the phenotype of an offspring is also influenced as long as the environmental stress is maintained (Crill et al. 1996; Magiafoglou and Hoffmann 2003; Bonduriansky and Head 2007). So, one can assume that as long as the stress is maintained no one can discriminate acclimation from adaptation process as changes in the phenotype of the offspring could persist. After stopping the stress if phenotype changes are canceled (reversible changes) one can conclude that acclimation process were involved nevertheless, if these changes are not canceled no one can discriminate acclimation from adaptation process.

Moreover, previous approaches described in the literature have involved subjecting one microbial generation to a progressive heating (heat slope) (Martínez de Marañón et al. 1999; Guyot et al. 2005; Morozov and Petin 2007). Guyot et al. (2010) showed that cells of *E. coli* that had been exposed to a heat slope had a higher survival rate than heat-shocked cells and so, acclimation could be involved in this type of thermotolerance.

This study is intended to know if a slow and continuous increase in temperature allows bacterial cells to acclimate to a temperature higher than the known upper limit of their thermal niche in a fewer number of generations than that previously described (i.e., more than 600). Thus, fed-batch cultivation of *E. coli* exposed to a very slow rate of heating for 9.5 days was performed. The two strains of *E. coli* examined were a well-described laboratory strain (*E. coli* K12-TG1) and an environmental strain (*E. coli* DPD3084), which has recently been thermally characterized (Guyot et al. 2010). These strains were grown in a fed-batch medium by daily renewing 90% of the growth medium which has led to propagate bacteria and to appreciate eventual growth at high temperature in an environment of continuously increasing temperature at a rate of 1°C 12 h^−1^, from 37°C to the temperature at which total cell death occurred. Although bacteria were propagated for only 30 generations during the slow rate of heating, our results showed a great increase in growth and survival thermal limits, respectively, until 48–50°C and 52–54°C. To characterize the mechanisms involved in cell survival at high temperatures, the bacterial cells collected after the slow-warming process were (1) subjected to a cooling step and a subsequent heat stress (which allowed to evaluate the reversibility of the acquired thermotolerant mechanisms); and (2) structurally analyzed (membrane and proteins) using Fourier transform infrared (FTIR) spectroscopy. The results showed the prevalence and the rapidity of acclimation phenomena and the involvement of membrane fluidity and protein aggregation.

## Material and Methods

### Bacterial strains and culture conditions

The bacterial strains *E. coli* K12-TG1 and *E. coli* DPD3084 (provided by DuPont, Wilmington) were used in this study. The strain K12-TG1 is a wild-type strain whereas the strain DPD3084 is genetically modified with a grpE::luxCDABE fusion at the chromosomal *lacZ* locus (Van Dyk et al. 2000). Although this genetic characteristic (i.e., luminescent signal generation by luciferase synthesis under the control of the *grpE* promoter) was not used in this study, this strain is of great interest because it has been thermally characterized well in a recent study (Guyot et al. 2010).

Colonies of *E. coli* were plated on petri dishes containing Luria–Bertani (LB) medium (25 gL^−1^) agar (13 gL^−1^) (Sigma, Saint Quentin Fallavier, France). Precultures were prepared by transferring a single colony into a 250 mL conical flask containing 100 mL of LB broth. The precultures were then shaken at 200 rpm overnight at 37°C on a temperature-controlled shaker (C24-KC, New Brunswick Scientific, Edison, NJ).

#### Slow warming

The cultures were prepared as follows: 100 *μ*L of preculture was used to inoculate 100 mL of similar growth medium. The cultures were shaken at 200 rpm for 24 h, and then grown in a fed-batch medium. Every 24 h, 90 mL of the existing culture was removed and 90 mL of fresh medium, prewarmed to the desired temperature, was added to the 10 mL of remaining culture. To apply slow warming, the target temperature was changed every 12 h at a heating rate of 1°C 12 h^−1^ in the range of 37–56°C.

Temperature was controlled using a K thermocouple (TCSA) immersed in the liquid medium. The experiments were performed in triplicate, at least, using three independent precultures. Each group consisted of control cultures grown at 37°C and stressed cultures subjected to slow warming, both initiated from the same preculture.

### Enumeration of bacterial populations

Cells were enumerated with a colony-forming unit (CFU) assay. Ten-fold serial dilutions of the cell suspensions were made by introducing 100 *μ*L of these suspensions into 900 *μ*L of growth medium, and then at least three 10 *μ*L drops of the appropriate dilutions were plated individually on petri dishes containing 15 mL of LB–agar medium. All petri dishes were incubated at 37°C for 24 h, regardless of the temperature at which the samples were harvested.

### Determination of the upper limit of the thermal niche

The maximal growth temperature was measured for each strain using the CFU method. Briefly, as previously described by Guyot et al. (2010), 2 mL of washed culture was rapidly (within no more than 2 sec) introduced into 50 mL of medium prewarmed to the following temperatures: 42, 44, 45, 46, 48, 50, 53, and 54°C (heat shock). The cell cultures were then maintained at the desired temperature for 2 h. The cells were counted at 0, 1, and 2 h. The experiments were performed on three independent cultures for each strain. The relative percentage of cell count was defined as the ratio of the number of CFU in the heat-shocked sample divided by the number of CFU in the corresponding unshocked sample (i.e., the control) multiplied by 100. Temperature was controlled using a K thermocouple (TCSA) immersed in the liquid medium. The results are given as means, and the 95% confidence intervals of the means were calculated.

### Testing the hypothesis of reversible/irreversible thermotolerance induced by slow warming: Exposure of cells to rapid cooling and then to a heating step

Aliquots (1 mL) of slowly warmed cells and the corresponding control cells were collected after incubation for 5.5 days at 47 and 37°C, respectively, and then inoculated into 100 mL of fresh growth medium introduced into a 250 mL conical flask maintained at 37°C. The cultures were (1) placed in a rotary incubator shaker (New Brunswick Scientific) for 15 min at 37°C with agitation (200 rpm); and then (2) transferred to a second rotary incubator shaker (New Brunswick Scientific) prewarmed to 47°C, and shaken at 200 rpm. The cell suspensions were maintained for 1 h at 47°C. The cells were counted after 10 sec and 1 h at 47°C. The number of colonies (CFU) per drop was measured and the percentage of cells present after 1 h at 47°C relative to that after 10 sec (100%) was calculated. The results are given as means, and the 95% confidence intervals of the means were calculated.

### Evaluation of protein aggregation and membrane fluidity using infrared spectroscopy

The changes in protein structure and membrane fluidity were estimated using FTIR spectroscopy. The spectra were measured using hydrated (and not dried) samples to evaluate the aggregation of proteins and changes in lipid fluidity. Aliquots (5 mL) of the control and slowly warmed cells were centrifuged at 2880*g* for 5 min at 37°C. The cell pellets were then washed twice in 100 *μ*L of ^2^H_2_O (Sigma) by centrifugation at 2880*g* for 5 min at 37°C (so the samples were cooled to 37°C for 15 min during the centrifugation steps and this cooling step was taken into account when the results were analyzed and discussed). The washed cell pellets were then placed on an infrared-transparent ZnSe window and equilibrated for 5 min at the desired temperature (47°C). Note that an additional set of cell suspensions was heat treated at 47°C for 5 min and analyzed directly on the FTIR machine. The temperature was regulated by water circulation in a double envelope surrounding the window. All the attenuated total reflectance FTIR second-derivative spectra were recorded between 4000 and 900 cm^−1^ (wavenumbers) on a Vector 22 FTIR spectrometer from Bruker (Karlsruhe, Germany) with a Bio-ATR II unit and equipped with a mercury–cadmium–telluride (MCT) detector. The spectral resolution was 4 cm^−1^. To obtain the spectrum for each sample, 10 scans were recorded and analyzed using the OPUS 6.5 software (Bruker). Several data preprocessing algorithms were used to analyze all the FTIR spectra. Data preprocessing first included a smoothing of the spectrum (17 smoothing points, provided by the OPUS 6.5 software) and then a second-derivative transformation. To evaluate protein aggregation and membrane fluidity, the second-derivative spectra were normalized with respect to the corresponding tyrosine band (around 1515 cm^−1^) or the CH_3_ asymmetric stretching band (around 2956 cm^−1^). Normalization against a specific band (tyrosine or CH_3_) meant that the intensity of this band could be used as the internal standard and was set to unity. The peaks of interest were determined using the OPUS 6.5 peak-picking function: only peaks with negative intensities were considered “true” peaks.

To evaluate protein aggregation, the characteristics (in terms of intensity and wavenumber) of the intermolecular *β*-sheet bands close to 1632 cm^−1^ (Doglia et al. 2008) and 1695 cm^−1^ (Gonzalez-Montalban et al. 2006) (i.e., *β*-sheets in aggregates) were measured. A shift from about 1632 cm^−1^ to close to about 1620 cm^−1^ indicated that the *β*-sheets were more condensed (García-Fruitós et al. 2007).

Variations in plasma membrane fluidity were evaluated by measuring the vibrational modes of the (*v*_*α*_) CH_2_ symmetric stretching band located around 2850 cm^−1^ (Leslie et al. 1995; Mille et al. 2002).

## Results

### Measurement of the upper limits of the thermal niches

**Figure 1:**
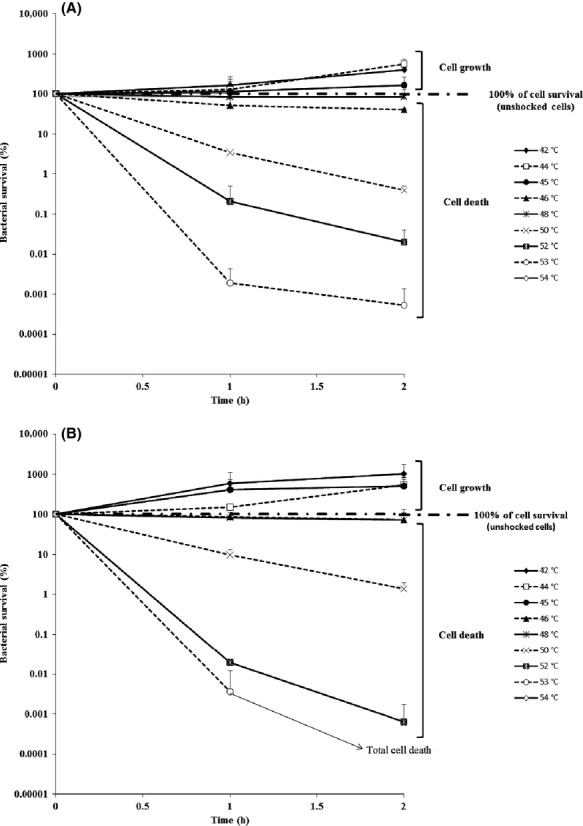
Determination of the upper limit of the thermal niches of two *E. coli* strains. Bacterial cells were heat shocked to different temperatures ranging from 42 to 54°C. Cell survival of the strains (A) *E. coli* DPD3084 and (B) *E. coli* K12-TG1 was measured after a 1-and 2-h plateau phase at the desired temperature. The relative percentage of cell count was defined as the ratio of the number of CFU in the heat-shocked sample divided by the number of CFU in the corresponding unshocked sample (i.e., the control = 100%) multiplied by 100. The upper limit of the thermal niche corresponds to the maximal temperature at which 100% bacterial survival is recorded whereas survival rate higher and lower than 100% correspond, respectively, to cell growth and cell death. Note that no points are represented for 54°C because no cell survived. The means of at least three independent measurements were calculated and the 95% confidence intervals of the means are presented.

As explained in the Material and Methods section, the bacterial cells were heat shocked at 42, 44, 45, 46, 48, 50, 53, or 54°C for 2 h. The survival curves are presented in Figure 1 where the percentage is related to the number of viable cells counted before the thermal treatment was applied (i.e., control = 100% of cell survival). So, the upper limit of the thermal niche corresponds to the maximal temperature at which 100% bacterial survival is recorded whereas survival rates higher and lower than 100% correspond, respectively, to cell growth and cell death. The survival curves, presented in Figure 1A (strain DPD3084) and 1B (strain K12-TG1), showed that the upper limits of the thermal niches were between 45 and 46°C for the two strains. Indeed, cell growth occurred after 2 h at 45°C (cell survival was about 163.1% for strain DPD3084 and about 516.9% for strain K12-TG1), whereas cell death occurred at 46–48°C for DPD3084 (40.5% and 84.1% of cells survived after 2 h, respectively, at 46°C and 48°C) and at 46°C for K12-TG1 (71.9% of *E. coli* K12-TG1 cells survived after 2 h at 46°C). Whatever the strain, total cell death was recorded at 54°C. No cells may be able to grow between 2 h and 24 h at 48°C and 53°C meaning that the initial decline recorded at these temperatures was due to death and that no later grow could occur. Indeed, about 41% and 8% of, respectively, *E. coli* DPD3084 and K12-TG1 cells survived after 24 h at 48°C and no cells survived after 2 and 24 h at 53°C whatever the strain considered. So, there were slight differences in the thermal growth/survival behavior of the two strains.

### Measurement of the upper thermal range of cell growth and the upper thermal range of cell survival during slow warming

**Figure 2:**
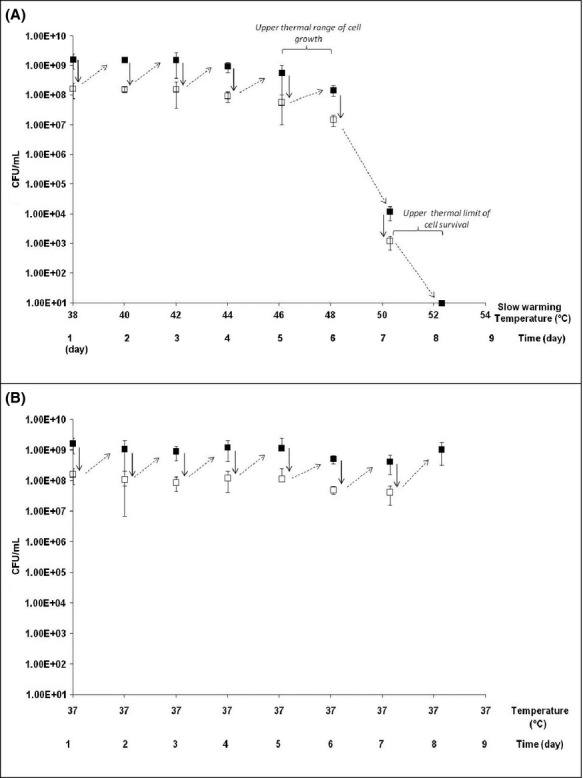
Evolution of bacterial populations according to temperature and time. The present results were obtained using the strain *E. coli* DPD3084 (repetition no. 3 from Table 1). CFU counts were made (A) during slow warming with temperature/time, and (B) during a plateau phase at 37°C (control). During slow warming, the temperature target was changed every 12 h at a heating rate of 1°C 12 h^−1^. (▪) the CFUs of the bacterial populations were measured at each time point during the heat-stress treatment. (→) every 24 h, 90 mL was removed from the 100 mL bacterial suspensions and replaced with 90 mL of fresh medium; thus, (□) every 24 h, a decimal dilution of the suspension was achieved. (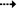) represents the general evolution of the bacterial population (and not the exact kinetics) during the 24-h period between two decimal dilutions. Averages of at least three measurements were calculated, bars represent the 95% confidence intervals of the means.

The CFU numbers of five bacterial populations of *E. coli* DPD3084 and three bacterial populations of *E. coli* K12-TG1 were measured as slow warming was applied from 37 to 56°C at a rate of 1°C 12 h^−1^. An example of the CFU counts for *E. coli* DPD3084 over time and temperature is shown in Figure 2. As previously explained (see Material and Methods section), a decimal dilution of the suspension was made every 24 h, as shown by the dotted lines in Figure 2. The solid lines represent bacterial growth or death during the 24 h between two successive decimal dilutions. We also confirmed the similar growth behaviors of the bacterial suspensions maintained at 37°C (i.e., control) between two successive decimal dilutions to validate the method (Fig. 2).

To compare the behaviors of the two strains, their upper thermal ranges of cell growth and the upper thermal ranges of cell survival are presented in Table 1. These parameters were evaluated between two decimal dilutions. If the cell counts increased with temperature after a decimal dilution, it can be concluded that cell growth occurred, whereas if the cell counts decreased with increasing temperature after a decimal dilution, it can be concluded that cell growth was abolished and the cells merely survived (the number of surviving cells was measured after the decimal dilutions). The results presented in Table 1 show that bacterial growth occurred until at least 44–46°C. Surprisingly, the upper thermal range of growth for *E. coli* strain DPD3084 varied in the range of 44–50°C (five repetitions), whereas that for *E. coli* strain K12-TG1 was 44–46°C. Our results also show that the upper thermal range of cell survival was mainly 48–50°C for strain DPD3084 and 46–48°C for strain K12-TG1. The cell counts made before total cell death was observed (i.e., 0.00 CFU mL^−1^) varied in the ranges of 9.33 × 10^1^–4.68 × 10^9^ CFU mL^−1^ for strain DPD3084 and 1.00 × 10^1^–1.35 × 10^5^ CFU mL^−1^ for strain K12-TG1. Strain DPD3084 showed an interesting behavior in that one bacterial population (repetition no. 5) survived up to 52–54°C and 1.28 × 10^3^ CFU mL^−1^ were present 24 h before total cell death. This result shows that this population survived for at least 24 h in the temperature range 52–54°C.

**Table 1:** Determination of the upper thermal range of cell growth and the upper thermal range of cell survival during slow warming.

Strain	Repetition	Upper thermal range of cell growth		Upper thermal limit of cell survival	
		Temperature (°C)	Cell counting (CFU mL^−1^)	Temperature (°C)	Cell counting (CFU mL^−1^)
*E. coli* DPD3084	1	44–46	3.50 × 10^7^	48–50	3.83 × 10^3^
	2	44–46	3.40 × 10^8^	48–50	9.33 × 10^2^
	3^1^	46–48	1.53 × 10^8^	50–52	1.23 × 10^4^
	4	46–48	9.50 × 10^7^	48–50	1.25 × 10^3^
	5	48–50	4.88 × 10^10^	52–54	1.28 × 10^3^
	A	44–46	3.73 × 10^6^	46–48	1.00 × 10^2^
*E. coli* K12-TG1	B	44–46	3.80 × 10^7^	46–48	1.35 × 10^6^
	C	44–46	3.23 × 10^7^	46–48	4.50 × 10^2^

1 Evolution of bacterial population from repetition no. 3 (*E. coli* DPD 3084) according to temperature and time is presented in Figure 2A.

In light of these observations, it appears that this slow warming allowed the bacterial cells of *E. coli* to grow in the temperature range of 44–50°C, which is in the same order of magnitude or higher than the upper limit of the thermal niches of the two strains (i.e., 45–46°C; see Fig. 1). However, in some cases, this thermal treatment allowed the preservation of a great degree of cell survival after 24 h in the temperature range of 48–50°C, which is outside the thermal niche (i.e., dramatic cell death was measured after 1 and 2 h heat-shock treatments; see Fig. 1). Thus, during slow warming, the bacterial cells expressed thermotolerance mechanisms. The nature of these mechanisms attracted our interest because not all cell populations displayed the same behavior in terms of cell counts and thermal limits. From these observations, we can ask whether these mechanisms are related to the acclimation process. To answer this question, we checked the reversibility of this thermotolerance with temperature variations because such reversibility would confirm the involvement of an acclimation mechanism. We focused on strain DPD3084, which showed a higher level of thermotolerance than strain K12-TG1.

### Experimental tests of the acclimation assumption

**Figure 3:**
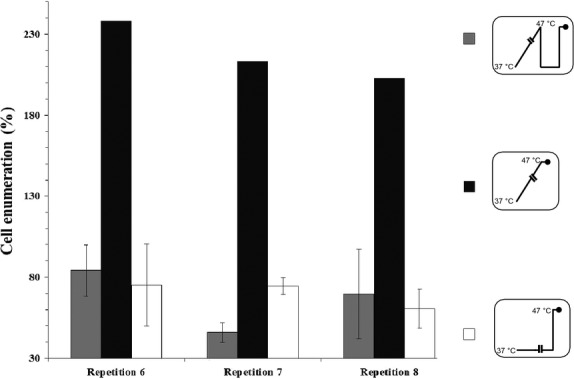
Measurement of cell enumeration of *E. coli* DPD3084 collected at 47°C, cooled to 37°C, and then exposed to a heating step at 47°C. (□) Corresponding control cells were collected at 37°C (after 5.5 days) and then heat shocked at 47°C for 1 h; (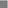) slowly warmed cells (repetitions 6, 7, and 8) were collected at 47°C (after 5.5 days), and then both were maintained at 37°C for 15 min and finally exposed to a heating step at 47°C for 10 sec or 1 h. Bars represent the 95% confidence intervals of the means. (▪) Represents the expected enumeration of slowly warmed cells maintained for 1 h at 47°C (in the absence of a cooling step to 37°C). The results were calculated as the percentage of cells counted after 1 h at 47°C relative to those counted after 10 sec (100%). Insets represent the temperature variations as a function of time, where the black circles indicate measurements.

Because reversible phenotypic changes refer necessarily to acclimation, we can assume that a cooling step from the temperature reached during slow warming (47°C) to the initial growth temperature (37°C) could cancel the mechanisms involved in thermotolerance (because these were associated with the capacity of the bacteria to survive at 47°C). To characterize these mechanisms, slowly warmed bacterial cells (repetitions 6, 7, and 8) were harvested at 47°C (after 5.5 days), then maintained at 37°C for 15 min, and finally exposed to heating to 47°C for 1 h. These cultures were chosen because they were characterized by an upper thermal range of growth higher than 46–48°C. The percentage of cell enumeration after 1 h at 47°C was calculated relative to that after 10 sec (100%) and presented in Figure 3 (gray histograms). Enumeration of the previously slowly warmed cells (collected at 47°C) was compared with that of the cells grown to 47°C during slow warming (Fig. 3, black histograms) and that of the control cells (grew at 37°C and then heat shocked at 47°C for 1 h (Fig. 3, white histograms).

First, the results presented in Figure 3 show that after 1 h at 47°C, enumeration of slowly warmed cells from repetitions 6 to 8 (previously cooled from 47 to 37°C for 15 min, gray histograms) was slightly decreased (at least 15% of cells died between 10 sec and 1 h at 47°C), whereas more than 50% of the cells from repetition 7 died. Second, our results show that enumeration of cells collected after slow warming to 47°C/subsequent cooling to 37°C for 15 min/and a heat shock at 47°C for 1 h (gray histograms) was dramatically lower than that expected (more than 200%, black histograms; for each repetition, *P* ≤ 4.10^−7^). This observation indicates that the cooling step from 47 to 37°C canceled the capacity of the slowly warmed cells to grow at 47°C. The loss of such a capacity was confirmed by comparing enumeration of slowly warmed cells subsequently cooled and heat shocked (gray histograms) to that of the control cells heat shocked at 47°C for 1 h (white histograms). Indeed, for repetitions 6 and 8 after the heat shock at 47°C the level of cell death in control cells (about 20%) was similar to that of slowly warmed cells cooled and then heat shocked (*P* ≥ 0.1) whereas for repetition 7 the level of cell survival of control cells (about 74%) was significantly higher than this of slowly warmed cells subsequently cooled and heated (about 45%; *P* < 2.10^−8^). Thus, slow warming did not allow the bacterial cells to maintain a greater capacity to survive at a high temperature than their ancestral cells (i.e., the control cells).

This result led us to conclude that the mechanisms involved in the maintenance of bacterial division at 47°C were reversible and not compatible with the adaptation assumption. So, acclimation process is necessarily involved in this type of thermotolerance.

Therefore, we hypothesize that such reversible mechanisms could be related to passive ones (as rapid and reversible changes in plasma membrane fluidity) and not to reversible changes with long duration persistence as cumulative/active ones like the persisting presence of chaperone synthesized during the slow warming and potentially active during the recovery at 37°C (Veinger et al. 1998; Tomoyasu et al. 2001). To test the possible involvement of such rapid reversible mechanisms (and so of acclimation process with short duration persistence) in this type of thermotolerance, plasma membrane fluidity variations of slowly warmed cells collected at 47°C, cooled to 37°C, and finally heat stressed at 47°C were analyzed using FTIR spectroscopy. Because fluidity of plasma membrane phospholipids change with temperature, such a cooling step should be sufficient to cancel any changes occurring during the slow warming. Moreover, using the same approach we also appreciated changes in protein conformation in order to verify that no protein aggregation/denaturation occurred with temperature increase and persist even in the presence of the subsequent cooling step.

### Spectroscopic characterization of membranes and proteins after a cooling step followed by a heating step

To characterize the effects of a cooling step followed by a heating step on the physicochemical structures as plasma membrane fluidity and protein aggregation of slowly warmed bacteria, three bacterial cultures of *E. coli* DPD3084 (repetitions 6, 7, and 8) were exposed to slow warming and then analyzed using a FTIR method. Slowly warmed cells were collected at 37°C on the 1st day (control cells) and at 47°C at 5.5 days. The cells were first centrifuged at 37°C for 15 min (which mimicked the cooling step applied at 37°C for 15 min) and were then analyzed at the temperature at which they were collected (i.e., slowly warmed cells were analyzed at 47°C, which corresponded to the subsequent heating step).

#### Plasma membrane fluidity changes

The changes in the fluidity of the plasma membrane were determined by measuring the changes in the vibrational modes of the wavenumbers of the symmetric (*ν*_*s*_) CH_2_ stretching bands located at about 2850–2858 cm^−1^. The measurements were monitored at 47°C and the second-derivative spectra normalized against the CH_3_ asymmetric stretching band were studied. The results are presented in Table 2 where Δ represents the difference between the considered heat-treated sample and its corresponding control. If Δ is negative, one can assume that the plasma membrane fluidity of the heat-treated sample is lower than that of the control and inversely. Our results show that at 47°C, the slowly warmed cells (subsequently cooled to 37°C for 15 min and then heat treated at 47°C/5 min) presented negative values of Δ (Table 2A) whereas heat-shocked cells presented positive values (Table 2B) meaning that after a heat shock the plasma membrane of cells previously exposed to a slow warming was less fluid than that of cells directly heat shocked. Because the fluidity of a membrane is related to its composition, one can assume that the composition of the plasma membrane of slowly warmed cells subsequently cooled to 37°C and analyzed at 47°C was different from that of immediately heat-shocked ones at 47°C. Thus, one can ask whether plasma membrane chemical and physical states changed during the slow warming meaning that homeoviscous adaptation of the membrane occurred. Nevertheless, the fluidity parameter seemed not to be a key factor related to cell survival as similar level of cell enumeration was recorded after a slow warming followed by a cooling step then a heat shock and a sole heat shock (Fig. 3).

**Table 2:** Spectroscopic analysis of (*ν*_*α*_) CH_2_ vibration and relative intensity of *E. coli* DPD3084 at 47°C.

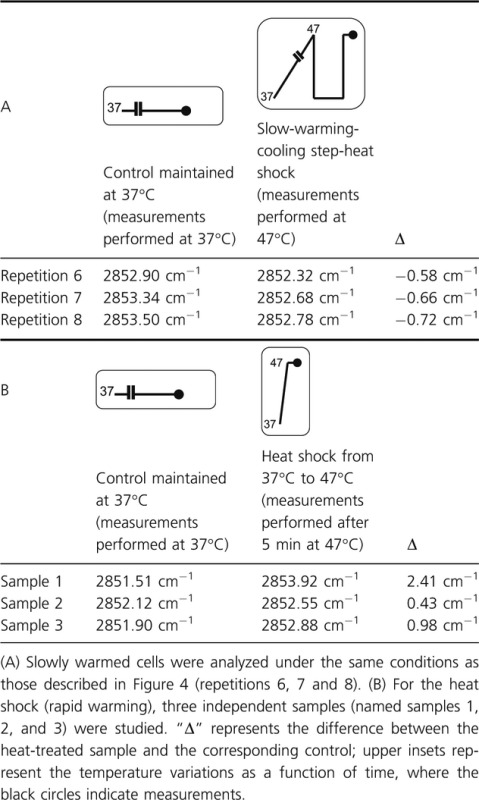

#### Protein conformational changes

**Figure 4:**
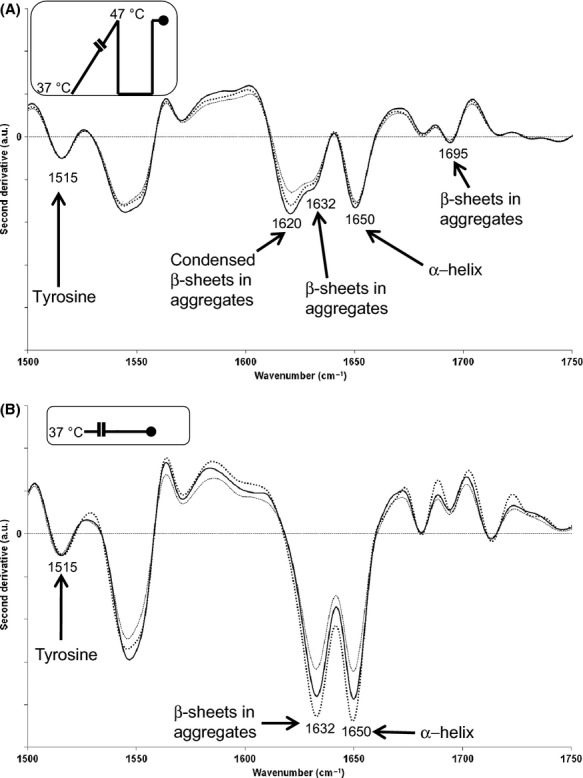
Spectroscopic analysis of protein aggregation in slowly warmed and heat-treated bacterial cells of *E. coli* DPD3084. Secondary protein structure was analyzed with Fourier transform infrared spectroscopy (FTIR) Second-derivative spectra were normalized against the tyrosine band (around 1515 cm^−1^) in the range of 1500–1750 cm^−1^. Second-derivative spectra of (A) slowly warmed cells (repetitions 6–8), and (B) the corresponding control cells were obtained using raw spectra measured at 47 and at 37°C, respectively, after a centrifugation step at 37°C for 15 min. In (A) and (B), the numbers represent wavenumbers (in cm^−1^) and the corresponding chemical functions are indicated with arrows. Insets represent the temperature variations as a function of time, where the black circles indicate measurements.

Variations in the protein conformational changes were evaluated using second-derivative spectra ranging from 1500 to 1750 cm^−1^ and normalized against the tyrosine band. The results are presented in Figure 4. The second-derivative spectra of the cells slowly warmed to 47°C, then cooled to 37°C for 15 min (centrifugation steps), and analyzed at 47°C are presented in Figure 4A and show a downshift of the *β*-sheets in the aggregate band from about 1632 cm^−1^ to about 1620 cm^−1^, which can be attributed to the formation of condensed *β*-sheets. Moreover, a shoulder at around 1695 cm^−1^, attributable to *β*-sheets in the aggregates was also observed. Interestingly, in the control cells (grown and analyzed at 37°C, Fig. 4B), no peak at 1620 cm^−1^ and no shoulder around 1695 cm^−1^ was observed. These observations led us to conclude that the level of protein aggregation was higher in the slowly warmed cells than in the control cells. Nevertheless, these changes did not severely alter cell survival at this temperature.

## Discussion

The slow warming improved bacterial survival and growth at a high level of temperature through acclimation process. Our results obtained with slow rate of heating showed that cell growth can be preserved until 50°C and cell survival until 54°C (Fig. 1). Such kinetics allowed the cells to survive for 2 days in a very high temperature range, which has not previously been reported (Table 1). Interestingly, all the repetitions of *E. coli* K12-TG1 but not those of *E. coli* DPD3084 exhibit the same thermotolerant behavior in the case of slow warming (Table 1). Most studies have described the growth of *E. coli* cells over a large number of generations within a range of high temperatures: either only slightly higher than the thermal niche (19–42°C) (Bennett and Lenski 1993) or largely higher at 48.5°C, as described by Rudolph et al. (2010). However, in accordance with Chown et al. (2009) and Mitchell and Hoffmann (2010), who investigated animal thermotolerance under ramping conditions, our observations suggest that the value of the upper limit of the thermal niche also depends on the protocol used. So, to compare results obtained by different laboratories, the protocols must be standardized and one can ask whether the best protocol should be based on the one that confers the highest upper thermal limit. Any such protocol must allow organisms to maximally express their phenotypic plasticity potential.

To verify the hypothetical acclimation mechanisms involved in cell survival during slow warming, the slowly warmed bacterial cells (from 37°C to 47°C within 5.5 days) were subjected to a rapid cooling at 37°C, then maintained at 37°C for 15 min, and finally exposed to a heating step at 47°C. This approach allowed us to assess the persistence or reversal of those mechanisms.

The results showed a great reduction in the cell counts, similar to that in the control cells suddenly heated at 47°C. So, the cooling step canceled the capacity of the slowly warmed cells to grow at 47°C which confirmed the acclimation hypothesis. So, this protocol brings simple evidence to acclimation occurrence. Obviously, adaptive mechanisms cannot be involved here because (1) the bacterial cells were propagated over only a few generations (about 30 generations), and (2) all the repetitions exhibit a thermotolerant behavior in the case of slow warming (Table 1). Indeed, beneficial mutations generally appear over a large number of generations which could be different from a repetition to another. For instance, Philippe et al. (2007) reported that only 10 mutations (not the expected pattern for beneficial mutations) were discovered in 12 populations propagated over 10,000 and 20,000 generations. The extremely probable lack of beneficial mutations after only 30 generations is also consistent with the previous observations of Bennett and Lenski (1997), who acclimated bacteria to different thermal environments (from 27°C to 40°C) for only 6–7 generations before measuring their fitness because of cross-generational effects. In light of these considerations, we can already suggest that the thermotolerance acquired during slow warming is attributable to acclimation mechanisms (phenotypic acclimation) and not to genetic adaptation.

### Slow warming induced bacterial thermal acclimation over a few generations

Interestingly, this study showed that it is possible to acclimate *E. coli* to very high temperatures (i.e., 54°C) through only 30 generations. Although bacteria were propagated at a temperature higher than the upper temperature limit during only 3 days (from 46°C so, over 10–12 generations), we hypothesize that acclimation process could start when bacteria reached 40°C (so over about 6 days and less than 19 generations) because (1) when cells were rapidly heat shocked at 46°C (Fig. 1) for 2 h no growth was recorded and (2) previous studies showed that *E. coli* cells can be acclimated in the range 39–46°C (Table 3). Moreover, previous works summarized in Table 3, were conducted at a lower level of temperature (i.e., 48.5°C) than the one reached here, showing that *E. coli* could be acclimated to over more than 620 generations. Our results are novel and demonstrate that acclimation is a rapid process with regard to time and so to the number of generations. Moreover, we hypothesize that the maintenance in such a state of acclimation could be useful to species during slow environmental changes because they could allow additional time to acquire incidental beneficial mutations.

**Table 3:** Comparison of thermal data for different strains of *Escherichia coli*.

Growth temperature of the ancestor	Number of generations propagated or propagation time at the upper thermal growth limit	Upper thermal growth limit	Upper thermal survival limit	Warming kinetics	*E. coli* strain	References
37°C	About 30 generations	48.0–50.0°C	52.0–54.0°C	Slow warming (1°C. 12 h^−1^)	DPD3084	This work
37°C	Less than 20 generations	44.0–46.0°C	46.0–48.0°C		K12-TG1	This work
37°C	620 generations	48.5.0°C	–	Stepwise increase in temperature^1^	MG1655	Rudolph et al. (2010)
37°C	–	47.4°C	–	Rapid warming	Nontypable strain	Salter et al. (1998)
30°C	–	45.8°C	–	Rapid warming	10 strains from a meat product	Membré et al. (2005)
37°C	Maintenance of the population in the face of 100-fold daily dilution	39.0–40.0°C	Death measured at 44.0°C	Rapid warming?	B	Mongold et al. (1999)
42°C		42.0°C				Mongold et al. (1999)
37°C	2000 generations	41.0°C	–	Rapid warming?	B	Bennett and Lenski (1993)
42°C		42°C				Bennett and Lenski (1993)

1 The stepwise increase in temperature did not correspond to a linear increase in the temperature according to time.

### Analysis of membrane fluidity and protein conformation confirmed the acclimation hypothesis

A complementary infrared spectroscopy analysis led us to appreciate changes in plasma membrane fluidity and protein conformation during the thermal cycle applied after the slow warming (i.e., a cooling step followed by a heat shock).

Our results strongly suggested that changes in plasma membrane fluidity were effectively involved in the slow-warming induced thermotolerance. Indeed, we observed that at 47°C, the slowly warmed cells (subsequently cooled to 37°C for 15 min and then heat treated at 47°C/5 min) presented lower level of plasma membrane fluidity than that of cells immediately heat shocked at 47°C (not exposed to a subsequent cooling step to 37°C) (Table 2). Our results suggest that a homeoviscous adaptation regulated the membrane fluidity during the slow warming in order to maintain membrane functionality. Complementary measurements (data not shown) based on the analysis of the (*v*_*α*_) CH_2_ sym/(*v*_*α*_) CH_3_ sym intensity ratio (Burattini et al. 2008; Cavagna et al. 2010) led us to suggest that the homeoviscous regulation of the plasma membrane was not related to changes in length of lipid chains. Thus, such an approach gave evidence to previous observation showing that slow-warming induced thermotolerance was related to acclimation process at the level of the plasma membrane. So, the alternative evidence given by long and fastidious complete genome sequencing is no more useful than such an approach.

Concerning proteins, we showed the persistence of aggregated proteins with condensed *β*-sheets in the aggregates (both in the control and slowly warmed cells; see Fig. 4A and B) and more particularly the presence of condensed *β*-sheets in aggregates (only in the slowly warmed cells; Fig. 4A). Because both control and slowly warmed cells were alive, protein aggregation cannot be related to any absence of protein activity as it was proposed by García-Fruitós et al. 2007.

This study has shown that slow warming applied over 9.5 days allowed mesophilic bacterial cells of *E. coli* to be propagated up to the very high temperature range of 48–54°C. Our results show that the degree of thermotolerance of the laboratory strain (K12-TG1) was lower than that of the environmental strain DPD3084. Interestingly, this thermotolerance, which was rapidly acquired over less than 19 generations, was shown to be related to reversible acclimation mechanisms and not to persisting adaptive mechanisms.

Moreover, this study strongly suggests that (1) the plasma membrane structure was not well-maintained after the heating and cooling steps, (2) the plasma membrane fluidity was a key factor in cell division when the slowly warmed cells reached 47°C and (3) the aggregation of proteins is compatible with cell survival. So, to acquire thermotolerance, bacteria must be subjected to a slow warming whose kinetics is adapted to the constant time of cellular. Future work will mainly focus in testing slower warming kinetics than that proposed in this study in order to know if the upper thermal limit of survival could be higher than that measured here and so if mesophilic bacteria are genetically programmed to rapidly acclimate to a slow and progressive temperature increase.

## Conflict of Interest

None declared.
